# Development and clinical evaluation of a CRISPR/Cas13a-based diagnostic test to detect *Mycobacterium tuberculosis* in clinical specimens

**DOI:** 10.3389/fmicb.2023.1117085

**Published:** 2023-02-03

**Authors:** Weicong Ren, You Zhou, Haoran Li, Yuanyuan Shang, Xuxia Zhang, Jinfeng Yuan, Shanshan Li, Chuanyou Li, Yu Pang

**Affiliations:** ^1^Department of Bacteriology and Immunology, Beijing Chest Hospital, Beijing Tuberculosis and Thoracic Tumor Research Institute, Capital Medical University, Beijing, China; ^2^Chest Hospital of Guangxi Zhuang Autonomous Region, Liuzhou, Guangxi, China; ^3^Department of Tuberculosis, Beijing Center for Disease Prevention and Control, Beijing, China

**Keywords:** tuberculosis, *Mycobacterium tuberculosis* complex (MTB), GeneXpert MTB/RIF, CRISPR, PCR, diagnosis

## Abstract

**Objective:**

Tuberculosis diagnosis requires rapid, simple and highly sensitive methods. Clustered regularly interspaced short palindromic repeats (CRISPRs) and associated protein (Cas) systems are increasingly being used for clinical diagnostic applications, due to their high flexibility, sensitivity and specificity. We developed a sensitive *Mycobacterium tuberculosis* (MTB) complex polymerase chain reaction (PCR)-CRISPR/Cas13a detection method (CRISPR-MTB) and then evaluated its performance in detecting MTB in clinical specimens.

**Methods:**

The conserved MTB IS1081 sequence was used to design CRISPR-derived RNAs (crRNAs) and T7 promoter sequencing-containing PCR primers for use in the CRISPR-MTB assay, then assay performance was evaluated using 401 clinical specimens.

**Results:**

The CRISPR-MTB assay provided a low limit of detection of 1 target sequence copy/μL and excellent specificity. Furthermore, use of the assay to detect MTB in bronchoalveolar lavage fluid (BALF), sputum and pus samples provided superior sensitivity (261/268, 97.4%) as compared to sensitivities of acid-fast bacilli (130/268, 48.5%) and mycobacterial culture (192/268, 71.6%) assays, and comparable or greater sensitivity to that of GeneXpert MTB/RIF (260/268, 97.0%).

**Conclusion:**

The CRISPR-MTB assay, which provides excellent sensitivity and specificity for MTB detection in sputum, BALF and pus samples, is a viable alternative to conventional tests used to diagnose TB in resource-limited settings.

## Introduction

Tuberculosis (TB), caused by *Mycobacterium tuberculosis* (MTB) complex, remains one of the leading infectious causes of death worldwide, with an estimated 1.6 million TB deaths in 2021 (World Health Organization, 2022). Rapid and accurate TB diagnosis is critical to ensure timely initiation of anti-TB therapy (Walzl et al., 2018). Nevertheless, 58.89% of the estimated 9.9 million new incident cases that emerge globally each year are undiagnosed. These undiagnosed cases act as a reservoir that fuels ongoing TB transmission that is hindering efforts towards meeting the ultimate goal of global TB eradication (Pai et al., 2016).

Currently, TB detection and diagnosis are based on clinical symptoms, radiologic abnormalities and laboratory test results. However, smear microscopy to detect tubercle bacilli, a century-old test that continues to be widely used for TB diagnosis worldwide, does not provide adequate sensitivity when used to test specimens obtained from children, HIV-infected individuals and extrapulmonary TB cases. Despite its status as a gold standard TB diagnostic test, mycobacterial culture testing of sputum specimens takes several weeks to complete, due to the intrinsic slow growth characteristics of tubercle bacilli (Miller et al., 1994). Hence, due to the abovementioned drawbacks of currently used tests, better tests are needed to facilitate early diagnosis of active TB cases. Recent technological advances have facilitated development of molecular diagnostic assays that have enabled more rapid, specific sensitive detection of *Mycobacterial tuberculosis* (MTB) bacilli in patient specimens. Several of these assays have been endorsed by the World Health Organization for use in initial diagnostic testing of suspected TB cases, such as Xpert MTB/RIF, Xpert MTB/RIF Ultra and loop-mediated isothermal amplification (TB-LAMP). However, the cost per test of such assays, which can be >20-times higher than that of smear microscopy, has hindered efforts to scale up such testing in low-income countries, warranting the development of less expensive and more accurate molecular diagnostic assays to identify active TB cases in resource-limited settings.

Clustered regularly interspaced short palindromic repeats (CRISPR) and associated protein (Cas) systems have been used increasingly for clinical diagnostic applications, due to their high flexibility, sensitivity and specificity (Yan et al., 2019). These novel methods can efficiently detect pathogens in human specimens by converting target nucleic acid sequences into fluorescent signals. For example, the CRISPR/Cas12a-based platform has already been used to diagnose TB and other infectious diseases (Xiao et al., 2020; Li et al., 2021; Qiu et al., 2021; Jiang et al., 2022; Lei et al., 2022). Meanwhile, another CRISPR/Cas system based on Cas13a, an RNA-guided RNA-targeting endonuclease, holds great promise as a molecular diagnostic platform. In fact, by combining Cas13a collateral RNase cleavage with molecular amplification, Zhang and colleagues established a CRISPR/Cas13a-based platform that has already been shown to detect specific viruses with attomolar-scale sensitivity and single-base specificity (Gootenberg et al., 2017; Kellner et al., 2019). However, to our knowledge, the diagnostic efficacy of this platform when used to detect tubercle bacilli has not yet been assessed in clinical practice.

In this study, we aimed to develop a CRISPR/Cas13-based diagnostic assay (hereafter referred to as CRISPR-MTB) for use in detecting MTB bacilli in TB patient specimens. The diagnostic performance of the proposed assay was then evaluated using multiple types of clinical specimens obtained from patients with TB and other respiratory diseases.

## Methods

### Study participants and collection of clinical specimens

A total of 401 participants, including 268 MTB patients (pulmonary TB and osseous TB) and 133 non-MTB group patients (lung cancer, non-TB infectious diseases of respiratory system) were recruited at Beijing Chest Hospital from August 2021 to May 2022. MTB group patients were diagnosed based on clinical symptoms suggestive of active TB plus positive evidence obtained from results of sputum smear testing that included acid-fast bacilli (AFB), GeneXpert MTB/RIF and mycobacterial culture assays and/or imaging findings. Meanwhile, samples were collected from enrolled patients with other respiratory diseases using the aforementioned assays or other microbiological tests. Genomic DNA was extracted from all clinical specimens and stored at −80°C for CRISPR-MTB testing.

### Ethics statement

This study was conducted according to tenets specified in the Declaration of Helsinki of the World Medical Association and approved by the Ethics committee of Beijing Chest Hospital, Capital Medical University (approval number: YJS-2021-0926). Patients or surrogates signed informed consent forms.

### Bacterial strains and human DNA

*Mycobacterium tuberculosis* H37Rv (ATCC27294), *M. bovis* Bacillus Calmette–Guérin (BCG), *M. kansasii, M. abscessus, M. avium, M. intracellulare, M. gordonae,* and *M. fortuitum* were maintained in our laboratory. *Escherichia coli* and *Streptomyces globisporus* were purchased from China General Microbiological Culture Collection Centre (CGMCC). Purified human DNA was purchased from Solarbio Co., Ltd. (Beijing, China) and dissolved in nuclease-free water.

### Cas13a protein and other reagents

Primers and plasmids used to clone the MTB target sequence were synthesised by Sangon Co. Ltd. (Shanghai, China). The Cas13a protein used in this study, LwCas13a, was expressed and purified according to instructions provided by Professor Zhang of the Academy of Military Sciences Beijing, China (Gootenberg et al., 2017). LwCas13a protein was quantified according to instructions provided with the Bradford Protein Assay Kit (Beyotime Biotechnology, Shanghai, China). Aliquots of purified LwCas13a protein were stored at −80°C.

### Nucleic acid and crRNAs preparation

The conserved MTB insertion element IS1081 that was selected for use as the target of the MTB-detection assay was cloned into the pUC57 vector to construct recombinant plasmids. After recombinant plasmids were confirmed as correct *via* sequencing, they were purified using an EasyPure® HiPure Plasmid MaxiPrep Kit (TransGen Biotech, China). Next, plasmid DNA was quantified based on optical density measurements conducted at 260 nm, then DNA copy number was calculated using the formula (6.02 × 10^23^) × (ng/μl × 10^−9^)/(DNA length × 660) = DNA copy number/μl. The crRNA template was an 84-base-pair single-stranded DNA (ssDNA) consisting of the T7 promoter sequence, repeat sequence and target sequence. To prepare crRNA, double-stranded DNA (dsDNA) was amplified using the 84-base-pair ssDNA template and its flanking primers (Supplementary Table S1). After DNA was extracted with TRIzol Reagent (Invitrogen), the dsDNA product was transcribed overnight at 37°C to generate crRNA using the HiScribe® T7 Quick High Yield RNA Synthesis Kit (New England Biolabs). Thereafter, the transcription product was treated with DNase I at 37°C for 30 min to degrade the dsDNA template, then the final crRNA product was purified using an Agencourt RNAClean XP kit (Beckman Coulter) according to the manufacturer’s instructions.

### CRISPR-MTB assay

The CRISPR-MTB assay incorporated a PCR amplification step and a subsequent Cas13a detection step. The PCR mixture contained 25 μl 2× MightyAmp® Buffer Ver.3, 0.3 μM IS1081 sense (5′-ACAAAGCTTTCCAAGTCGCA-3′) and 0.3 μM IS1081 antisense (5′-AATTCTAATACGACTCACTATAGGGCCCA GGATCTCTCGGTAGC-3′) primers, 1 μl MightyAmp® DNA Polymerase Ver.3 and 2 μl of DNA sample in a total volume of 50 μl (adjusted with ddH_2_O). The PCR amplification programme consisted of denaturation at 95°C for 5 min, followed by 36 cycles (98°C for 10 s, 68°C for 20 s and 1 cycle at 68°C for 5 min) and generated a 237-base-pair amplicon. Cas13a detection was conducted using a reaction mixture containing the following constituents: 0.5 μl of murine RNase inhibitor (New England Biolabs), 45 nM purified LwCas13a, 45 nM crRNA, 125 nM quenched fluorescent RNA reporter (RNAse Alert, Thermo Scientific, Waltham, MA, United States), 0.5 μl of T7 polymerase mix, 1 mM ATP, 1 mM GTP, 1 mM UTP, 1 mM CTP and 2 μl of PCR product in nuclease assay buffer (40 mM Tris–HCl, 60 mM NaCl, 6 mM MgCl2, pH 7.3). Assays were carried out at 37°C for 30 min and monitored for a fluorescent signal using an ABI 7500 instrument (Thermo Fisher, MA, United States). Fluorescein (FAM) fluorescence values were read every 1 min.

### Genomic DNA extraction

Sputum was decontaminated with *N*-acetyl-l-cysteine (NALC)-NaOH and suspended as much as possible using a vortex mixer and then was incubated at 37°C for 30 min. Next, 1 ml of treated sputum or other type of sample was transferred to a nuclease-free, sterile 15-ml polypropylene tube. After centrifugation at 8,000 rpm for 10 min, the supernatant was removed and the pellet was washed twice in 2 ml of PBS buffer. Thereafter, the pellet was transferred to a new 1.5-ml tube and resuspended in 50 μl of PBS buffer. Next, tubes were heated at 100°C for 10 min and then were shaken at 1,500 rpm using a Thermo shaker followed by centrifugation for 10 min at 12,000 rpm. For PCR, 2 μl of extracted DNA of each sample served as template.

### Statistical analysis

All statistical analyses were performed using SPSS version 20.0 (IBM Corp., Armonk, NY, United States). All figures were created using GraphPad Prism 8 (GraphPad Software, Inc., CA, United States). Continuous variables were expressed as median (range) and categorical variables were expressed as percent (%) values. Student’s *t-*test, Mann–Whitney *U* test, Fisher’s exact test and Chi-square test were used to evaluate continuous and binomial variables. Intergroup differences were declared significant if two-sided *p-*values were less than 0.05. Statistically significant differences were presented as *p < 0.05, **p < 0.01, ***p < 0.001, and ****p < 0.0001.

## Results

### Development of the CRISPR/Ca13a-MTB assay

**Figure 1:**
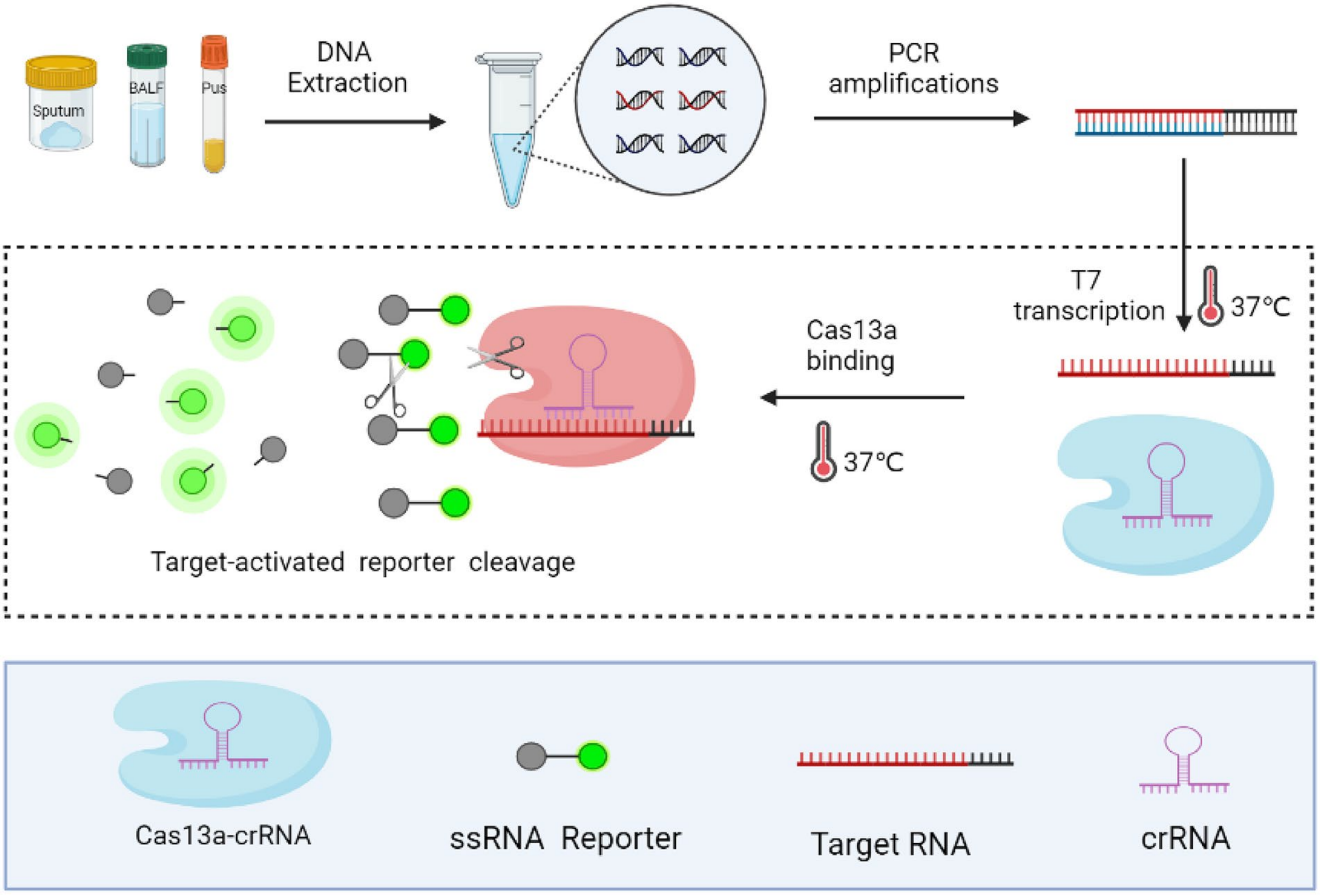
Schematic diagram of the CRISPR-MTB assay. Rapid DNA extraction from clinical specimens was performed followed by PCR amplification to increase the amount of target sequence, during which the T7 promoter sequence was attached to 5′ ends of PCR products. Next, PCR products were subjected to the CRISPR/Cas reaction, during which the collateral nuclease activity of Cas proteins was activated upon specific binding of crRNA to the MTB target gene sequence. Increased fluorescent signal produced by cleaved crRNA probes indicated the presence of MTB in clinical specimens.

We developed a highly sensitive and simple MTB-detection assay, with steps of this method presented in Figure 1. First, the target sequence (MTP IS1081) was amplified *via* PCR, during which the T7 promoter sequence was attached to 5′ ends of the PCR products. Next, the double-stranded DNA (dsDNA) amplicon was transcribed to generate single-stranded RNA (ssRNA) using T7 RNA polymerase. Thereafter, the ssRNA, under guidance of crRNA, was recognised and bound by Cas13a, which triggered collateral RNase cleavage of the reporter RNA molecule that resulted in release of fluorescent groups into the reaction solution. Significantly enhanced fluorescence indicated the presence of the target gene.

**Figure 2:**
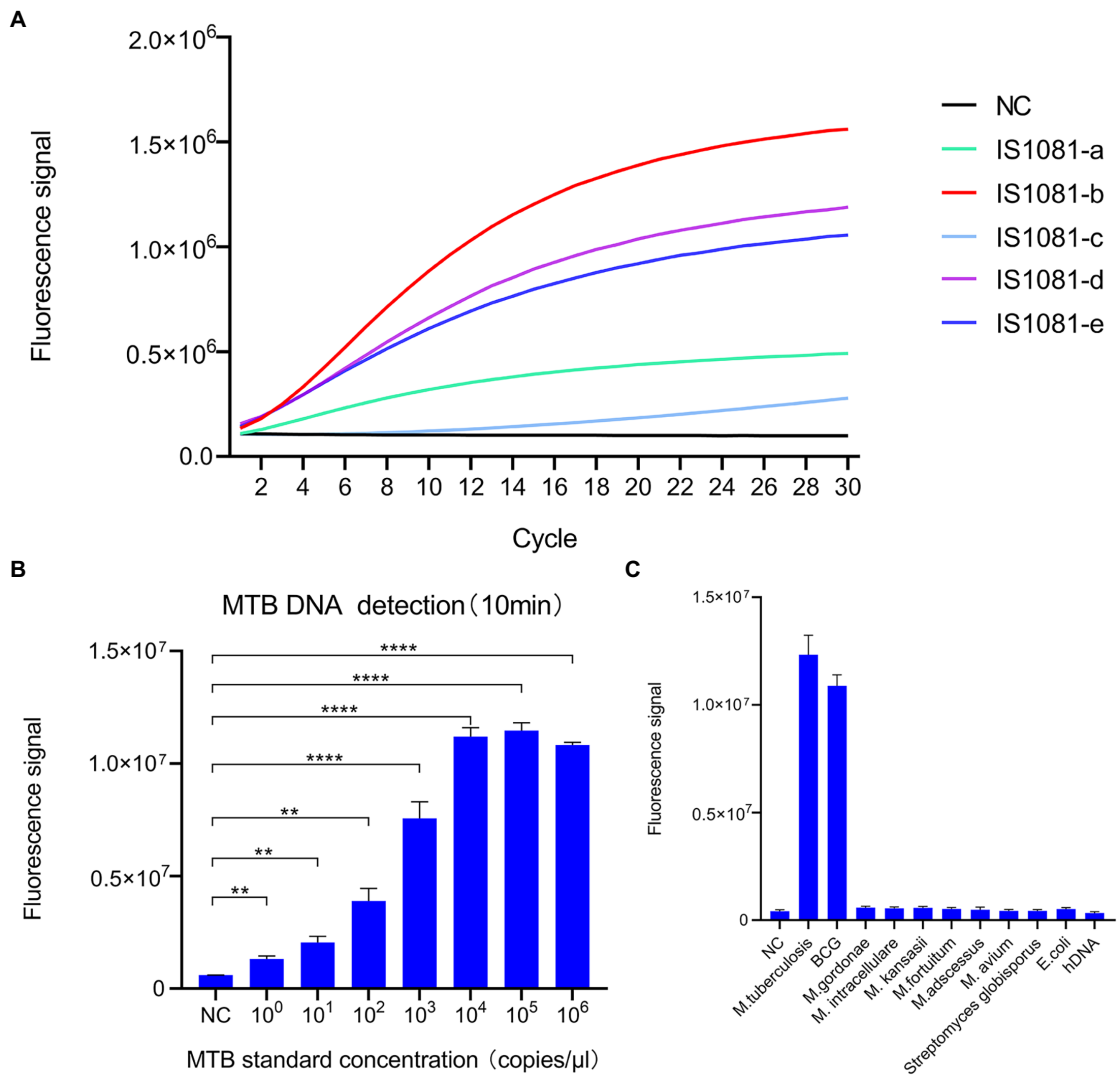
Development of the CRISPR-MTB assay. **(A)** Screening of crRNAs for MTB DNA detection ability based on Cas13a-induced detection. The signal obtained for the crRNA probe IS1081-b was greater than signals of all other crRNAs. **(B)** Analytical assessment of CRISPR-MTB sensitivity. CRISPR-MTB could detect one copy of MTB DNA in 10 min (*n* = 3 technical replicates; **p < 0.01, ***p < 0.001, ****p < 0.0001; bars represent mean ± standard error of the mean). **(C)** Analytical assessment of CRISPR-MTB specificity. Only genomic DNA of MTB and BCG were explicitly detected at 30  min, while genomic DNA samples without MTB or BCG produced no obvious signal (*n* = 3 technical replicates; bars represent mean ± standard error of the mean).

We selected the MTB-specific insertion sequence IS1081 as the target sequence, since this insertion sequence is present in multiple copies per genome, thus improving MTB-detection sensitivity. We then screened five candidate crRNAs (Supplementary Table S2) that targeted different rIS1081 sequences in order to select the crRNAs that produced the most intense fluorescence signal, as assessed using an ABI7500 fluorescence detector. The results (Figure 2A) revealed that the IS1081-b crRNA candidate provided greater fluorescence signal strength than signal strengths of the other four crRNAs.

In order to determine the analytical sensitivity of the CRISPR/Cas13a assay, a plasmid containing the IS1081 insertion sequence was used as a template, then the template was serially diluted to generate dilutions for limit of detection (LOD) evaluation. The results showed that an MTB DNA copy number as low as one copy per test could be detected after 10 min of PCR amplification using the IS1081-b crRNA (Figure 2B).

To confirm assay specificity, genomic DNA from human cells, *M. tuberculosis* (MTB) isolates and the MTB BCG strain, nontuberculous mycobacteria (NTM, including *M. kansasii, M. abscessus, M. avium, M. intracellulare, M. gordonae,* and *M. fortuitum*) or other bacteria (*E. coli*, *S. globisporus*) were tested using the CRISPR-MTB assay. As shown in Figure 2C, only MTB and BCG DNA contained the IS1081 target sequence; thus testing of samples containing only these organisms were the only samples that produced positive detection results. Altogether, these results suggest that CRISPR-MTB is a promising sensitive and specific molecular diagnostic assay for use in MTB detection.

### Detection of clinical TB cases using CRISPR/Ca13a-MTB

In order to further evaluate the diagnostic potential of the CRISPR-MTB assay for testing of clinical samples, 401 clinical samples were obtained from 268 TB cases (112 BALF, 141 sputum, and 15 pus specimens) and 133 non-TB cases (114 BALF and 19 sputum specimens) (Supplementary Table S3). Statistical analysis of patient characteristics revealed age differences between patients with and without active TB infections (Supplementary Table S3).

**Figure 3:**
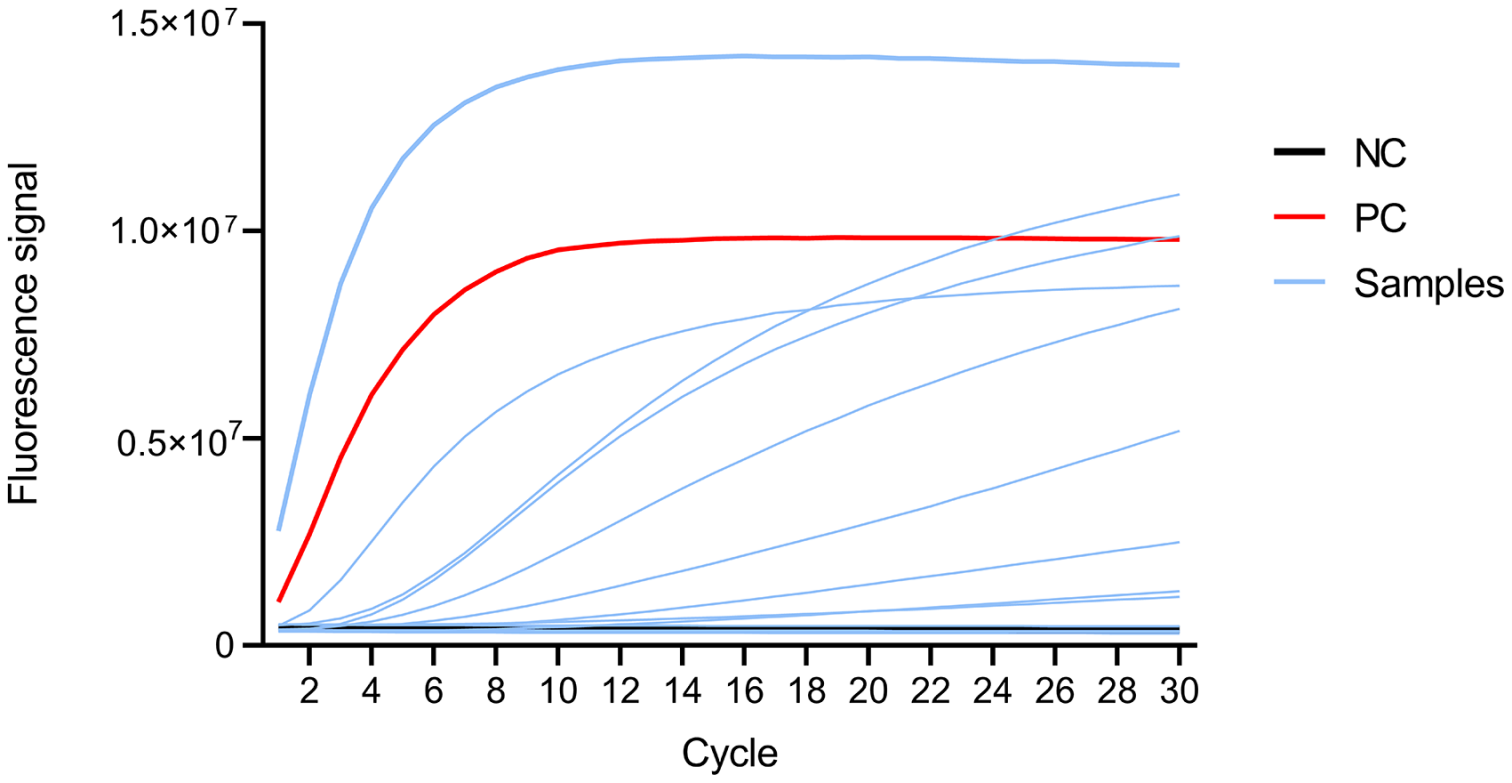
Fluorescence signal profile as detected using an ABI 7500 instrument. Black, red, and blue lines represent fluorescent signals from the negative control, positive control and samples, respectively.

**Figure 4:**
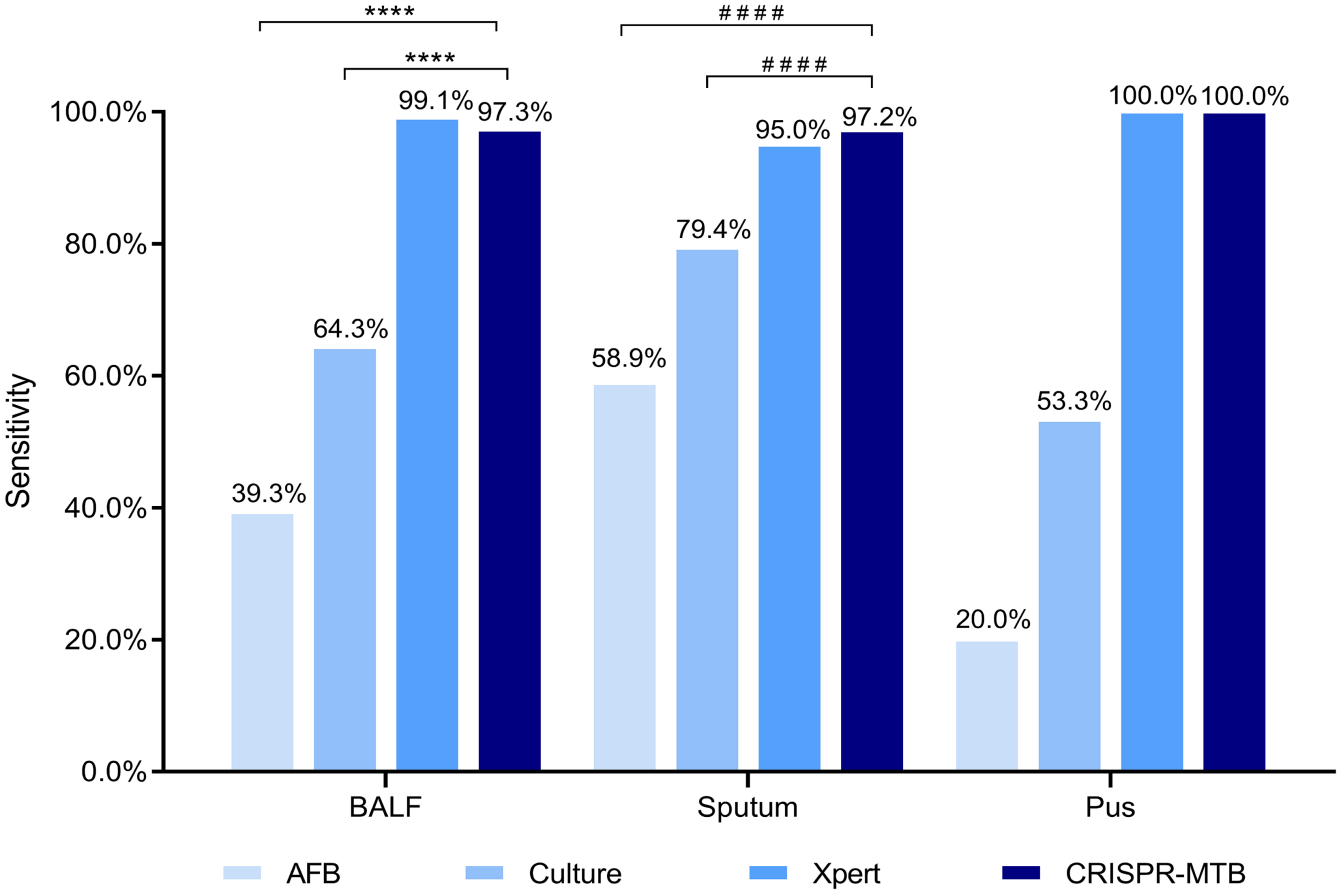
Evaluation of CRISPR-MTB sensitivity according to clinical sample type. Two hundred and twenty-six BALF, 160 sputum and 15 pus specimens were tested were detected *via* AFB, Culture, Xpert and CRISPR-MTB assays; ****McNemar test, *p* < 0.0001; ^####^Fisher’s exact test, *p* < 0.0001.

CRISPR-MTB assay results of control samples containing no template or plasmid DNA containing the target sequence served as the negative control (NC) and positive control (PC), respectively (Figure 3). Based on the final clinical diagnoses of patients as a reference, we evaluated CRISPR-MTB TB diagnostic performance for all patients in our study cohort (Supplementary Tables S4, S5; Figures 3, 4), and then compared MTB-detection results obtained *via* Xpert, culture, AFB and CRISPR-MTB assays for all samples. The results revealed that CRISPR-MTB assay TB diagnostic sensitivity was as high as 97.4% (261/268), which was higher than sensitivities obtained for the mycobacterial culture assay (71.6%, 192/268) and the AFB assay (48.5%, 130/268), and was at least as sensitive as that obtained for GeneXpert MTB/RIF (97.0%, 260/268), Hence, these results suggest that CRISPR-MTB is a highly sensitive TB diagnostic assay.

**Figure 5:**
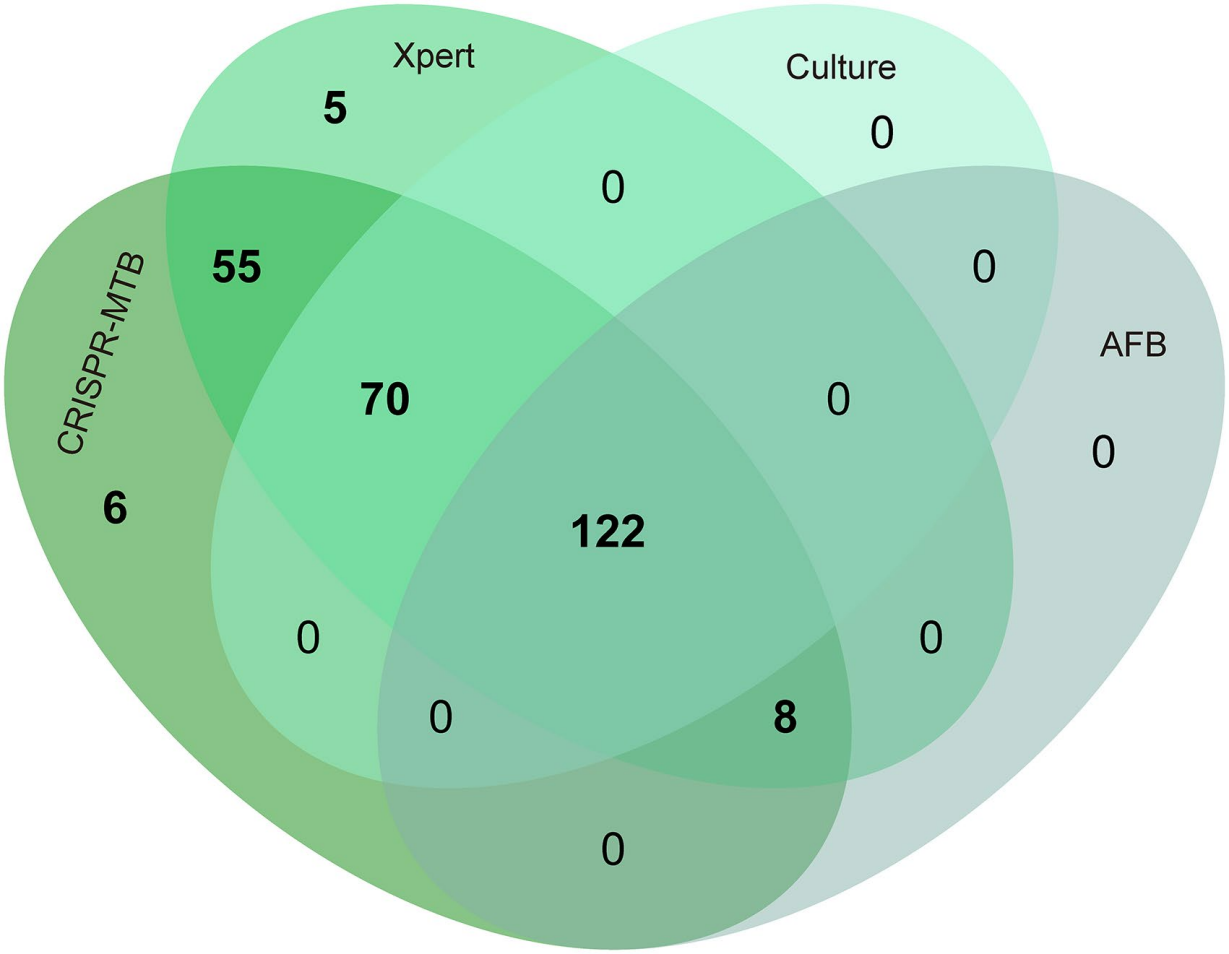
Venn diagram showing overlap in TB diagnostic test results obtained for different clinical MTB-detection assays.

After further analysis of the influence of clinical specimen type on assay diagnostic performance (Supplementary Tables S4, S5; Figure 4), it was found that CRISPR-MTB provided the highest sensitivity (97.2%, 137/141) when used to test sputum samples from pulmonary TB cases, which was significantly higher than that of AFB (58.9%, 83/141, *p* < 0.001) and mycobacterial culture (79.4%, 112/141, *p* < 0.001), and comparable to that of Xpert (95.0%, 134/141, *p* = 0.45). Similarly, the detection results obtained for BALF samples collected from pulmonary TB cases showed that CRISPR-MTB sensitivity reached 97.3% (109/112), which was higher than that obtained *via* mycobacterial culture (64.3%, 72/112), significantly higher than that obtained *via* AFB (39.3%, 44/112, *p* < 0.001) and statistically similar to that obtained *via* Xpert (99.1%, 111/112, *p* = 1.0). Meanwhile, testing of pus samples obtained from osseous TB cases *via* both CRISPR-MTB and Xpert detected all TB cases and significantly outperformed both MTB culture (100% vs. 53.3%) and AFB (100% vs. 20.0%) (Figure 4). Interestingly, CRISPR-MTB testing identified six clinically verified TB patients who would not have otherwise received a TB diagnosis based solely on their AFB and mycobacterial culture test results (Figure 5).

In addition, it is noteworthy that the CRISPR-MTB assay produced false-positive results for samples from six patients without TB infections (specificity: 95.5%, 127/133), which included patients with malignancies, non-TB infections and non-infectious inflammatory diseases, as compared to specificities of Xpert and culture assays (97.7 and 99.2%, respectively) (Supplementary Tables S4, S6). Nevertheless, these results collectively suggest that CRISPR/Cas13a-MTB provides excellent sensitivity and specificity for MTB detection, warranting further development of the assay for use in clinical TB diagnosis.

## Discussion

The discovery of CRISPR/Cas systems has generated a wave of development of innovative diagnostics that take advantage of both the sensitivity of PCR amplification and the specificity of the CRISPR system. In this study, we successfully developed a CRISPR/Cas13a-based diagnostic assay for use in diagnosing TB-based on clinical specimens. The limit of detection (LOD) of our assay (1 copy/μL) was below that reported recently by Ai and colleagues for a CRISPR/Cas12a-based system of 5 copies/μL of MTB DNA, indicating slightly greater sensitivity of our approach (Ai et al., 2019). Additionally, the TB-QUICK MTB-detection assay, which is based on CRISPR-Cas12b and loop-mediated isothermal amplification (LAMP), had a LOD that was as low as 1.3 copies/μL within 2 h, although the exact explanation for this result is unclear. Nevertheless, we speculate that the lower LOD obtained for the CRISPR-MTB assay was mainly due to the production of more precisely targeted transcripts during the T7 RNA polymerase step. However, of greater concern is the greater rate of false-positive results obtained with our assay, which may have been due to its ultra-high sensitivity. Regardless, our primary results demonstrated that the CRISPR-MTB assay provided excellent specificity, since no false-positive results were obtained for any of the six NTM-containing samples or for samples containing *E. coli* and *S. globisporus* species, due to the excellent single-base mismatch specificity of Cas13a (Chen et al., 2019). According to a previous study, as compared with mycobacterial culture testing, the gold standard clinical diagnostic method, the CRISPR-MTB assay developed here provided diagnostic sensitivity of 97.4% and specificity of 95.5%, while the sensitivity and specificity of Xpert were 75.9 and 82.8%, respectively (Moussa et al., 2016), and the sensitivity and specificity of CRISPR/Cas12a-based test were 79.0 and 98.0%, respectively (Ai et al., 2019). Thus, the CRISPR/Cas13a-based diagnostic is a viable alternative to conventional assays for use in detecting MTB in clinical specimens with high sensitivity and specificity.

As consistent with our analytical test results, CRISPR-MTB assay sensitivity was significantly greater than assay sensitivities obtained using conventional microbiological methods (e.g., smear microscopy, mycobacterial culture). This distinction is particularly important when paucibacillary BALF and pus specimens from extrapulmonary TB cases are tested, thus highlighting CRISPR-MTB as an assay that may improve diagnosis of paucibacillary extrapulmonary TB. Specifically, the CRISPR-MTB assay required a much lower volume of body fluid (500 μl) than that required by conventional methods. Thus, this assay may be more appropriate for identifying tubercle bacilli in specimens obtained from paediatric TB and tuberculous meningitis cases. Our study also emphasises the advantages of CRISPR-based detection assays for use in detecting MTB in specimens with paucibacillary loads, since current molecular MTB-detection assays provide suboptimal results when used to test such specimens, warranting further study.

It is noteworthy that the CRISPR-MTB assay was at least as sensitive as Xpert when used to detect MTB in sputa and other specimens, a result that appears, at first glance, to contradict our results showing that CRISPR-MTB had a lower LOD than Xpert. However, diagnostic sensitivity greatly depends on both the efficiency of a molecular procedure and on sample preparation and DNA extraction efficiencies, such that the ostensibly better performance of the Xpert assay may reflect its more efficient standardized extraction protocol involving fractionation of mycobacteria *via* a pre-sonication step (Patel et al., 2013). This result is supported by results of previous clinical evaluations demonstrating that Xpert outperformed other molecular tests currently used for TB diagnosis. By contrast, the relatively low efficiency of the manual DNA extraction method used in our assay may have reduced CRISPR-MTB sensitivity, while Venter et al. reported that crude DNA extracted using the Xpert cartridge was suitable for MTBDRsl (*Mycobacterium Tuberculosis* Drug Resistance second line) assays and produced more accurate second-line DST results than other DNA extraction methods (Venter et al., 2017). Taken together, these results highlight the importance of using efficient DNA extraction procedures to boost sensitivity of molecular diagnostic assays.

When considering the clinical applicability of the CRISPR/Cas13a-MTB assay, all steps are easily implemented in most clinical settings, since they rely on routine PCR amplification procedures and universal ‘RNA reporter’ probes that can be detected using common fluorescence detectors. Moreover, the assay can be completed in only 2 h using relatively inexpensive instrumentation and, as a critically important advantage, at low cost. In fact, in this pilot study the direct cost of each CRISPR/Cas13a-MTB test was less than $2 USD, a cost comparable to that of smear microscopy. However, the CRISPR-MTB assay provides dramatically greater diagnostic accuracy and thus is a superior alternative to routine TB diagnostic assays used currently in resource-limited settings.

We must acknowledge several limitations of the present study. First, the performance of CRISPR/Cas13a-MTB was only assessed based on a limited sample size, which may have weakened the significance of our conclusion, warranting further validation of the assay through testing of a greater number of specimens obtained from a larger patient cohort. Second, previous studies have demonstrated the potential role of LAMP in improving diagnostic sensitivity as compared with conventional real-time PCR (Sam et al., 2021). Thus, integration of LAMP amplification within the CRISPR-Cas13a system will be investigated in the future towards the development of a cost-effective point-of-care (POC) MTB detection assay (Kaminski et al., 2021). Finally, due to the intrinsic ability of Cas13a to target single-stranded RNA, an *in vitro* T7 transcription step was incorporated within the CRISPR-MTB diagnostic assay that undoubtedly increased the turnaround time as compared with DNA-targeting CRISPR-Cas systems.

In conclusion, we have successfully developed a CRISPR/Cas13a-based diagnostic test (CRISPR-MTB) to detect MTB in clinical specimens. Our data demonstrate that the CRISPR-MTB assay provides excellent sensitivity and specificity for rapid identification of MTB in sputum, BALF and pus samples and thus should be suitable for use as a TB diagnostic assay in resource-limited settings.

## Data availability statement

The original contributions presented in the study are included in the article/Supplementary materials, further inquiries can be directed to the corresponding authors.

## Ethics statement

This study was approved by the Ethics Committee of Beijing Chest Hospital, Capital Medical University. The patients/participants provided their written informed consent to participate in this study.

## Author contributions

YP and CL: conceptualisation and methodology, writing — review and editing. WR, HL, YZ, and SL: formal analysis and investigation. YS, XZ, JY, and YZ: data curation. WR, YZ, and HL: writing — original draft preparation. YP and WR: funding acquisition. All authors contributed to the article and approved the submitted version.

## Funding

This work was supported by the Beijing Key Clinical Specialty Project (20201214), the Beijing Hospitals Authority Ascent Plan (DFL20191601), the Beijing Hospitals Authority Clinical Medicine Development of Special Funding (ZYLX202122), and the Scientific Research Project of Beijing Educational Committee (KM202010025001). The funders had no role in the study design, data collection, analysis, interpretation or writing of the report.

## Conflict of interest

The authors declare that the research was conducted in the absence of any commercial or financial relationships that could be construed as a potential conflict of interest.

## Publisher’s note

All claims expressed in this article are solely those of the authors and do not necessarily represent those of their affiliated organizations, or those of the publisher, the editors and the reviewers. Any product that may be evaluated in this article, or claim that may be made by its manufacturer, is not guaranteed or endorsed by the publisher.
